# The value of surgeon’s perception during transurethral resection of bladder tumors: can we trust in our eyes and experience to predict grade and staging?

**DOI:** 10.1097/j.pbj.0000000000000179

**Published:** 2022-09-09

**Authors:** Luís Vale, José Sousa, Pedro Abreu-Mendes, Pedro Vale, Nuno Dias, Paulo Dinis, Tiago Antunes-Lopes, João Silva

**Affiliations:** a Department of Urology, Centro Hospitalar Universitario São João, Porto, Portugal,; b Faculty of Medicine, University of Porto, Porto, Portugal,; c USF São Neutel, Chaves, Portugal.

**Keywords:** predictive value, surgeon’s perception, transurethral resection of bladder tumor, urinary bladder neoplasms

## Abstract

Transurethral resection of newly diagnosed bladder tumors (TURBT) is a hallmark ¡n the treatment of bladder cancer. We evaluated the surgeon capacity to predict bladder tumor stage (T), grade, and presence of muscular layer based upon cystoscopic characteristics during primary TURBT.

**Methods:**

Prospective study enrolling 100 consecutive patients undergoing primary TURBT for newly diagnosed bladder cancers. Cystoscop¡c tumor characteristics at the time of TURBT was evaluated by an urology senior and a resident regarding histological grade, invasion (T stage), and presence of muscular layer in the specimen. We analyzed the surgeon’s accuracy in predicting these parameters using the final histology as gold standard. In addition, the predictive capacity between seniors and residents was compared.

**Results:**

The resident’s arm correctly predicted tumor invasiveness in 76% of cases, while seniors correctly predicted 87% of cases. Regarding tumor grade, high grade cancer was reported in 78% of the specimens and 75% and 77% of them were correctly predicted by residents and seniors, respectively. Finally, 80% of the TURBT specimens had muscle representativeness. In nearly 75% of the cases, both resident and senior correctly predicted the TURBT resection depth (presence of detrusor muscle in the specimen). The positive predictive value for this parameter was 79% for the resident, and 81% for the senior, and the negative predictive value was 25% and 40%, respectively.

**Conclusion:**

The surgeon’s naked eye analysis showed a good, but limited predictive ability to detect non-muscle invasive and high-grade bladder tumors in TURBT specimens. Positive predictive value for muscle representativeness is around 80%, which reinforces the need of carrying out a careful and extensive TURBT, irrespective of the surgeon experience.

## Introduction

Bladder tumor is the sixth most diagnosed carcinoma in the male population worldwide.^1^ Transurethral resection of bladder tumor (TURBT) is considered an important tool for diagnosis and initial treatment.^2^ This procedure has three main goals: make the correct histological diagnosis, evaluate the tumor invasion, and remove all visible lesions.^3^ Additionally, the appearance during the initial evaluation in both cystoscopy and TURBT plays an important role in the decision-making process of the urologist. Attitudes like simple fulguration or the depth of resection depend heavily on the urologist’s impression during TURBT as well as the information that the urologist transmits to the patient, which will impact the understanding of the condition and the ability to make informed decisions.

TURBT is one of the most common operations in urology and considered by many as an “easy” surgery. However, several studies have reported that initial TURBT is incomplete in many cases, contributing to the high recurrence rate.^4^ Understaging is another risk in bladder tumors since muscle invasion is not identified during TURBT in 25% of invasive tumors.^5^ Therefore, early recurrence and incorrect staging are associated with inadequate resection of the initial tumor.^6^ The presence of detrusor muscle in the specimen is a key landmark of the quality of TURBT.^7^

To our knowledge, no study has yet evaluated any correlation between urologist’s perception during initial TURBT and the final pathology and comparing the impact of different levels of experience of the surgeon. Therefore, we aimed to assess the clinical prediction of bladder tumor stage and grade based solely upon cystoscopic appearances at the first TURBT and correlate these predictions with the final histology, as well as the capability for prediction the presence of detrusor muscle layer during the procedure.

## Material and methods

The study included 100 consecutive patients admitted to the urology department, of our tertiary care center, from September 2018 to December 2019, for management of newly diagnosed bladder tumor. The diagnosis of bladder cancer was based on flexible cystoscopy. Urine cytology was used as adjuvant to the diagnosis. Patients older than 18 years and with a suspicion of primary bladder cancer were included. Previous localized prostate cancer without signs of recurrence was allowed. On the other hand, previous diagnosis of bladder or upper tract urothelial cancer were exclusion criteria. Furthermore, patients with a positive cytology but a negative cystoscopy were not included.

Patients were submitted to the first TURBT, carried out by a urology resident with 2 to 5 years of experience. The procedure was supervised by a senior urologist, with at least 10 years of experience and specially dedicated to bladder cancer management. At the time of TURBT, surgeons had access to the patient digital file including the cystoscopy description of the lesion. White-light cystoscopy was used in all cases and bipolar was the preferred energy source.

During the TURBT, residents and seniors independently predicted muscle invasiveness (invasive or non-invasive), tumor grade (high or low), and detrusor muscle presence in the specimen (presence or absence). Data was recorded on a form.

One-hundred specimens were obtained and submitted to the pathology laboratory for routine histologic assessment, of which two did not present carcinoma. Data about muscle invasiveness, tumor grade, and the presence of detrusor muscle were collected in the pathology database. The pathologists in our institution used the 2004 World Health Organization (WHO) classification systems on all reports and so, invasive tumor in this report refers only to a muscle invasive (muscularis propria) bladder cancer.

Four patients were excluded because the resident and/or senior did not predict the grade, the invasiveness, or the presence of muscle in the specimen. After exclusion of pathology reports not explicitly stating one of the three parameters in study (3 cases), the final cohort included 91 TURBTs.

All statistical tests were performed using IBM SPSS Statistics for Windows, Version 25.0. (Armonk, NY: IBM Corp. Released 2017). The sensitivity, the specificity, the positive predictive value (PPV), and the negative predictive value (NPV) were calculated using a standard 2 × 2 table comparing the relative ratios of true/ false positives and negatives. The confidence intervals (CI) were produced with the Wilson Score method and statistical significance was defined as *P<* .05.^8^ The receiver operating characteristic (ROC) curve and the Fleiss’ kappa coefficient was used to describe the concordance between the clinicians’ prediction and the result of pathological anatomy. Agreement was classified into poor, fair, moderate, good, and very good reflecting Kappa values <0.20, 0.21-0.40, 0.41-0.60, 0.61-0.80, and 0.81-1.0, respectively.^9^

## Results

The pathological analysis revealed muscle invasive bladder tumors in 18.1% of the resections. The residents correctly predicted tumor invasiveness in 75.8% of cases, while the seniors correctly predicted 86.8% of the cases. Macroscopically, the sensitivity of the resident group’s impression to detect muscle invasive tumors was 82.4% and the specificity 73.3%, whereas the senior group had a sensitivity of 88.2% and a specificity of 87.7%. The positive predictive value (PPV) was 41.2% in the residents and 62.5% in the seniors, and the negative predictive value (NPV) was 94.8% and 97.0%, respectively.

Regarding the tumor grade, high-grade tumor was reported in 77.7% of the specimens. Of the 91 patients, 74.7% and 76.9% were correctly predicted based on visual appearance by the residents and the seniors, respectively. In this setting, the sensitivity and specificity of the residents were 84.3% and 42.9%, respectively, while those of the seniors were 85.5% and 55.0%. The PPV was 83.1% for residents and 86.8% for seniors, and the NPV was 45.0% and 52.4%, respectively.

Finally, 80.2% of the TURBT had muscle present in the specimen. In 73.6% and 75.8% of the cases, the resident and the senior group, respectively, correctly predicted that TURBT had been or not extensive enough to the detrusor muscle layer. The sensitivity of resident to report the presence of muscle in the specimen was 90.5%, and the sensitivity of senior was 90.0%; with a specificity of 11.8% for the resident and 23.5% for the senior. The PPV for this parameter was 79.2% for the resident and 80.6% for the senior, and the NPV was 25.0% and 40.0%, respectively.

Sensitivity, specificity, PPV, and NPV were summarized in Table 1, with 95% confidence intervals. There were no statistically significant differences in the three parameters.

**Table 1:** Predictive value for the parameters in study

Statistics	Resident Estimate value (%) [95% CI]	Senior Estimate value (%) [95% CI]
Muscle Invasive		
Sensitivity	82.4 [59.0–93.8]	88.2 [65.7–96.7]
Specificity	73.3 [62.4–82.0]	87.7 [78.2–93.4]
PPV	41.2 [26.4–57.8]	62.5 [42.7–78.8]
NPV	94.8 [85.9–98.2]	97.0 [89.6–99.2]
High grade		
Sensitivity	84.3 [74.0–91.0]	85.5 [75.3–91.9]
Specificity	42.9 [24.5–63.5]	55.0 [34.2–74.2]
PPV	83.1 [72.7–90.1]	86.8 [76.7–92.9]
NPV	45.0 [25.8–65.8]	52.4 [32.4–71.7]
Presence of detrusor muscle		
Sensitivity	90.5 [80.7–95.6]	90.0 [79.9–95.3]
Specificity	11.8 [3.3–34.3]	23.5 [9.6–47.3]
PPV	79.2 [68.4–86.9]	80.6 [69.6–88.3]
NPV	25.0 [7.1–59.1]	40.0 [16.8–68.7]

Statistical analysis for the three parameters in study: muscle invasiveness, tumor grade, and presence of muscle in the specimen. Sensitivity, specificity, positive predictive value [PPV], and negative predictive value [NPV] with 95% confidence interval [95% CI].

Using the Fleiss’ kappa coefficient (*k*), the agreement between the senior group and the pathologist, which was considered the gold standard, for muscle invasiveness was good (*k* = 0.653). Comparatively, the resident group only had moderate agreement with the pathologist for muscle invasiveness (*k*=0.376). Regarding the tumor grade, the agreement of both residents and seniors with the pathologist was fair (*k*=0.276 and *k*= 0.398, respectively). Concerning the presence of muscle in the specimens, the agreement of both groups with the pathologist was poor, and it was not possible to state that the agreement was better than chance (*k*=0.004 and *k*=0.147, respectively, with P > .05). Finally, we also decided to compare the agreement between the resident group and the senior group, which was fair to moderate. The statistical analysis regarding the agreement with the Fleiss’ kappa coefficient (*k*) was represented in Table 2.

**Table 2:** Agreement between groups

Fleiss’ kappa	Resident Estimate value [95% CI]	Senior Estimate value [95% CI]	Resident vs. senior Estimate value [95% CI]
Muscle invasiveness	0.376 [0.172–0.580]	0.653 [0.446–0.859]	0.491 [0.285–0.698]
Grade	0.276 [0.070–0.481]	0.398 [0.190–0.606]	0.440 [0.231–0.649]
Detrusor muscle	0.004 [–0.215–0.224]^*^	0.147 [–0.077–0.370]^*^	0.406 [0.184–0.628]

Fleiss’ kappa coefficient (*k*)with 95% confidence interval [95% CI]. The agreement between the resident group and the pathologist; the senior group and the pathologist; and the resident group and the senior group for the three parameters. P was <.05, except in^*^ cases.

**Figure 1.**
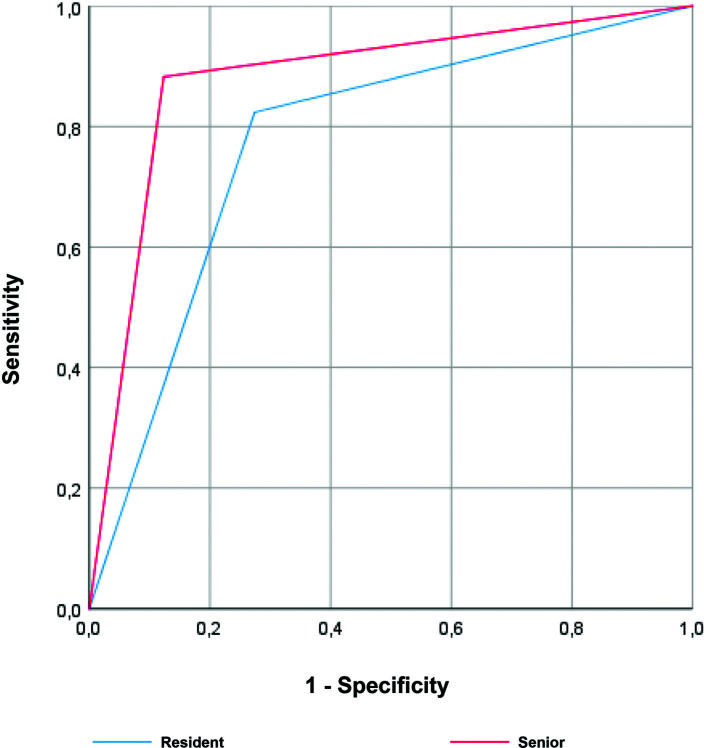
Receiver operating characteristic (ROC) curve for the ability of the resident (blue) and the senior (red) groups to detect muscle invasive tumors during primary TURBT. Area under the curve (AUC) for resident group was 0.775 (95% CI = 0.653-0.897), *P<* .05, while for senior group was 0.880 (95% CI = 0.780–0.979), *P <* .05.

**Figure 2.**
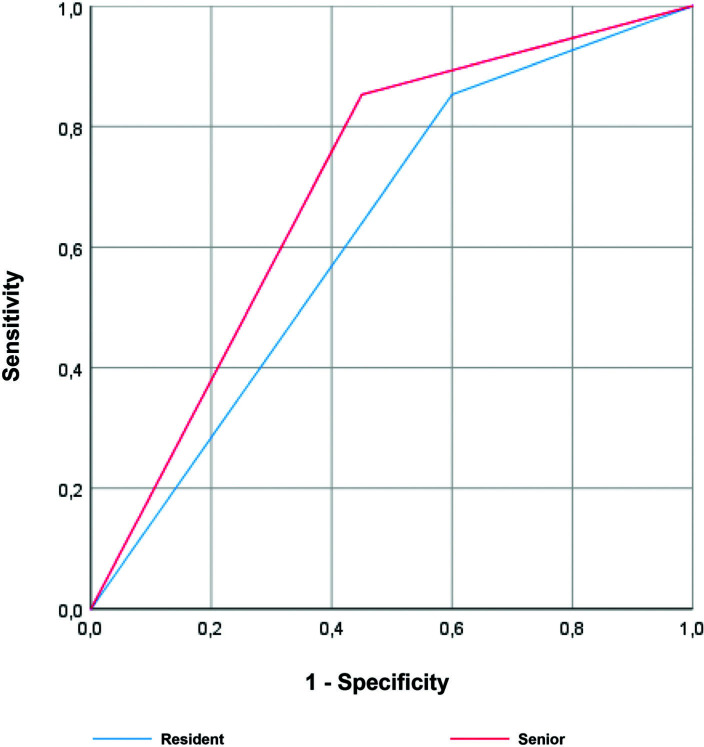
Receiver operating characteristic (ROC) curve for the ability of the resident (blue) and the senior (red) groups to detect high grade tumors during primary TURBT. Area under the curve (AUC) for resident group was 0.626 (95% CI = 0.477–0.775), *P* = .087, while for senior group was 0.701 (95% CI = 0.559–0.844), *P <* .05.

**Figure 3.**
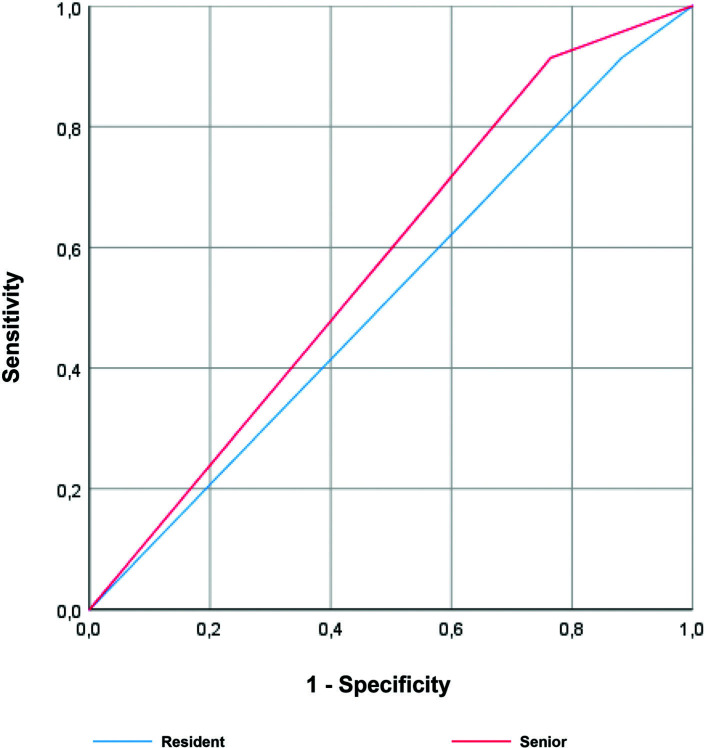
Receiver operating characteristic (ROC) curve for the ability of the resident (blue) and the senior (red) groups to detect the presence of muscle in the specimen during primary TURBT. Area under the curve (AUC) for resident group was 0.575 (95% CI = 0.411-0.738), *P* = .352, while for senior group was 0.616 (95% CI = 0.357–0.675), *P* = .844.

The accuracy of predicting invasive bladder tumor was described in the ROC curve, in Figure 1, a comparison between the resident group and the senior group. Both residents and seniors had high accuracy in predicting invasive carcinoma, although without statistically significant differences between the two groups. Regarding the grade, there was a tendency towards a higher area under the curve in the accuracy of seniors relatively to the residents, however without statistically significance (Fig. 2). Finally, through the ROC curve (Fig. 3), both residents and seniors showed no discrimination capacity to distinguish between the presence or absence of muscle in the specimen of the TURBT.

## Discussion

Urologists seem to have a great accuracy to differentiate clinically between high and low-grade tumors and predict muscle invasive disease with clinically reasonable accuracy in new bladder tumors.

The prediction performed by the urologist during TURBT is not being advocated as a replacement for the subsequent evaluation by the pathologist. However, this can be used as an immediate complement in the management of patients post-TURBT, while waiting for a definitive local staging.

The decision to perform an immediate intravesical single installation (SI) of chemotherapy should be based on the information available during TURBT, and to maximize effectiveness it should be administered within the first two hours after procedure.^11^ Not all patients benefit from SI. According to current guidelines, only patients with an European Organisation for Research and Treatment of Cancer (EORTC) recurrence score <5 benefits from SI. In the remaining patients, SI is not effective and is not recommended.^12^ The score is based on the number of tumors, size, prior recurrence rate, grade, stage, and concurrent CIS. Although the definitive histopathologic result is not yet known, the surgeon’s predictive ability can be used to estimate the final EORTC recurrence score.^12^ Certainly, this information would impact the surgeon’s decision to perform a SI.

Herr et al showed that urologist impressions of tumor stage and grade at cystoscopy correlated with the histology. Urologists correctly predicted the tumor stage and grade of 93% of Ta lowgrade tumors.^13^

Another more recent study suggests that visual assessment is accurate in predicting the presence of muscle invasion, and much more accurate in excluding it.^14^ The predictions through visual evaluation of muscle invasive bladder tumor presented a sensitivity of 88.9% and a specificity of 91.0%, giving a positive predictive value of 78.4% and a negative predictive value of 95.7%.^14^

Although TURBT is a very common procedure, the correlation between the urologist’s predictive ability and the final pathological findings is not well documented.

In this study, from the tumors that the senior considered non-muscle invasive, only 3.0% were invasive. Thus, the senior, with sensitivity and specificity of about 88.0%, has a good ability to confirm and exclude the invasiveness of the tumor. Moreover, the sensitivity of the senior for high-grade tumors is very similar to those of urinary cytology (85.5% and 84%, respectively).^15^ Although, less consistent results were obtained for the presence of detrusor muscle in the specimen, with low discriminatory capacity (Fig. 3). Both the resident and the senior demonstrated a good ability to identify specimens that presented detrusor muscle, with few false negatives (Table 1). In contrast, when the specimen had no detrusor muscle, both demonstrated a low capacity to confirm these (Table 1). These results reinforce the idea that TURBT is not an “easy” procedure and should be performed very carefully, broadly, and in-depth, since our impression about the presence of muscle layer is not very accurate.

TURBT is associated with a learning curve, so the inexperience and the lack of skills may adversely affect patient survival.^4^ Unsatisfactory pathology specimens may lead to additional procedures, exposing patients to additional surgical risks.^16^

Our results demonstrated that the best predictive ability of the urologist is for non-muscle invasive and high-grade tumors, while the worst is for the absence of muscle in the specimen. We also found that the resident’s predictive ability tended to be lower than that of the senior (not statistically significant), thus we cannot exclude that experience influences the surgeon’s predictive ability about primary bladder cancer stage and grade (Table 2).

The urologist’s predictive ability is limited by the visual identification of the tumor during TURBT, and by what the urologist considers to be a high- or low-grade tumor.^17^ Most urologists dealing with bladder tumor believe there are some tumor features, sometimes subtle (and somewhat subjective), that may be associated with tumor grade, but only acquired with experience.^10^

Nonetheless, we believe that the agreement was not as high as expected, because histologic examination of superficial bladder tumor remains difficult. Additionally, the general agreement between pathologists in staging and grading is 50% to 60%, which limits its prognostic value.^18^

With other techniques, it may be possible to increase the predictive ability of the urologist and improve patient management. For example, Blue Light Cystoscopy using hexaminole-vulinate increases detection rates of carcinoma in-situ (43%) and papillary lesions (12%) compared to White Light Cystoscopy alone and can change management in 14% of cases.^19^

Magnetic resonance imaging (MRI) can provide more information about the anatomy of the bladder, being useful in the preoperative diagnosis of bladder tumors.^20^ A recent meta-analysis has shown a sensitivity of 87% and a specificity of 79% to detect muscle invasive tumors.^21^ These results were very similar to those presented in our study for visual prediction during TURBT (88.2% and 87.7%, respectively), which may lead to considering the MRI as a complementary tool (or a replacement) for the second look.^21,22^

We acknowledge that a limitation of our study is the relatively few patients included; however, we hope that the present report stimulates further larger studies that may be of value in refining our clinical practice. Another apparent limitation is the small number that constitutes each group, seniors, and residents, which can interfere with external validity. Finally, future research should evaluate predictive factors of muscle invasion and combine it with the surgeon predictive ability to improve the detection of muscle invasion. This would avoid overzealous resection posing unnecessary risks of complications such as perforation or tumor spillage.

## Conclusion

According to our results, the surgeon’s naked eye analysis showed a good predictive ability to detect non-muscle invasive and high-grade bladder tumors in TURBT specimens. This information can impact the surgeon decision to perform SI after a first TURBT.

Our results also demonstrate that, although not statistically significant, seniors tended to have a higher predictive ability than the residents, thus we believe that experience can influence our predictive ability.

The positive predictive value for the presence of muscle in the specimen is around 80%. However, the ability to identify the presence of muscle layer in the specimen is poor, which reinforces the idea of carrying out an extensive and careful TURBT. Further studies are needed to compare the surgeon’s predictive ability for tumor parameters in the first TURBT with the results of the second look, to elucidate the real value of the second look in some cases.
